# Young children’s difficulties in switching from rhythm production to temporal interval production (>1 s)

**DOI:** 10.3389/fpsyg.2014.01346

**Published:** 2014-12-05

**Authors:** Anne Bobin-Bègue, Sylvie Droit-Volet, Joëlle Provasi

**Affiliations:** ^1^Laboratoire Éthologie, Cognition, Développement, Université Paris Ouest Nanterre La DéfenseNanterre, France; ^2^Clermont Université, Université Blaise Pascal, Laboratoire de Psychologie Sociale et Cognitive, CNRS UMR 6024Clermont-Ferrand, France; ^3^Laboratoire Cognition Humaine et Artificielle, École Pratique des Hautes ÉtudesParis, France

**Keywords:** timing, rhythm, sensorimotor synchronization, tempo, children

## Abstract

This study examined the young children’s abilities to switch from rhythm production, with short inter-tap intervals (ITIs), to temporal interval production, with long ITI (>1 s), in a sensorimotor synchronization task. Children aged 3- and 5-year-olds were given six sessions of synchronization. In a control group, they had to synchronize their ITI to an inter-stimulus interval (ISI) of 4 s. In the experimental group, they must progressively increase their ITI for one session to the next (from 0.4 to 4.0-s ISI). Our results showed that the 5-year-olds produced longer ITI that the 3-year-olds in synchronization. However, the value of ITI in the 5-year-olds never exceeded 1.5 s, with more variable ITI in the control than in the experimental group. In addition, at 5 years, boys had more difficulties than girls in changing their tapping rhythm. These results suggest a temporal window in sensorimotor synchronization, beyond which the rhythm is lost and the synchronization becomes difficult.

## INTRODUCTION

During childhood, one of the most important abilities is to efficiently coordinate actions with external events. This coordination requires timing abilities, i.e., estimating both event duration and the interval between two events, in order to produce actions at the right time, neither too late nor too early. The abilities involved in coordination of actions to external rhythms have been extensively studied in adults using a sensorimotor synchronization task (Repp, 2005; Repp and Su, 2013). The procedure used with children involves three phases (Drake et al., 2000; Provasi and Bobin-Bègue, 2003; Bobin-Bègue et al., 2006; McAuley et al., 2006; Bobin-Bègue and Provasi, 2008; Provasi et al., 2014). In the first phase [spontaneous motor tempo (SMT) phase], the participants are asked to tap with their finger on a button at their preferred rate, i.e., the most comfortable tapping rate. In the second phase (sensorimotor synchronization), they are asked to tap in synchrony with sounds displayed at a fixed inter-stimulus interval (ISI), generally shorter than 800 ms. Then, in a third phase (continuation phase), the auditory stimulus is removed and the participants are supposed to continue to tap at the same tempo. There are very few studies in young children using this sensorimotor synchronization task. However, some studies have found that the inter-tap interval (ITI) spontaneously produced by young children aged from 2 to 7 years of age lies between 400 and 500 ms but slows down during childhood to ∼600 ms in early adulthood (Drake et al., 2000; Provasi and Bobin-Bègue, 2003; McAuley et al., 2006; Bobin-Bègue and Provasi, 2008). More importantly, the variability (SD) of SMT decreases with age. During the sensorimotor synchronization phase, the variability in ITI is also greater in children than in adults (Repp, 2005). In addition, adults anticipate the auditory stimulus, thus showing a negative asynchrony between their finger tap and the beat of the metronome of about 10 ms (Miyake et al., 2004). However, this negative asynchrony is rarely found in young children. In fact, in the synchronization phase, very young children, aged from 1- to 3-years-olds, are able to synchronize their taps to an external auditory tempo, but only when it is close to their own SMT. Furthermore, in the continuation phase, they rapidly return to their initial SMT when the external auditory tempo is farther away from their SMT (Provasi and Bobin-Bègue, 2003; Bobin-Bègue and Provasi, 2008). These findings suggest that young children are able to synchronize their taps with external tempi but only when these are close to their SMT, i.e., to what Jones and Boltz (1989) call the referent period. Unlike children, adults succeed in producing longer ITI that their referent tempo up until a value of 1800 ms (Repp, 2005; Repp and Su, 2013). In sum, young children have a limited capacity for producing motor tempo outside their referent periods. This contributes to explain why adults adapt their rhythmic activities when interacting with infants (e.g., speaking more slowly; Gratier, 2003; Gratier and Trevarthen, 2008). However, it is possible that for children, tap synchronization with a dis-preferred tempo requires a lengthy learning period. It might thus be observable only with a greater number of trials and sessions than those used in previous studies. Consequently, the aim of the present study was to examine young children’s abilities to progressively learn to synchronize their motor tempo to external auditory tempi with longer ITI than their SMT.

According to Wing and Kristofferson (1973a,b) model, the total variance in sensorimotor synchronization results from addition of two sources of variance: a temporal variance related to central temporal processes (a central timekeeper) and a motor variance related to production of motor responses. The central timekeeper (clock) estimates the temporal interval between stimuli in a rhythmic sequence and determines when to initiate the response. The motor component is involved in the implementation of the motor response. Some authors (Keele et al., 1985; Ivry and Hazeltine, 1995) suggest that the temporal component involved in rhythmic activities with ITI in the 100 ms range is similar to that involved in the production of temporal intervals in the few seconds range. They thus posit the existence of a central timekeeper (clock) common to production of all temporal intervals regardless of their lengths. Whether there is a common clock system for the processing of short and long durations, we may predict that children can learn, over several training sessions, to produce longer ITI lasting several seconds in a sensorimotor synchronization task. Indeed, the motor component of synchronization, that is an easy task for young children, remains similar irrespective of different ISI (short/long). However, in the case of long ISI, we would describe their behavior as temporal interval production rather than sensorimotor synchronization because there is a duration limit beyond which sensorimotor synchronization no longer occurs. This limit is around 1800 ms (Fraisse, 1948; Repp, 2006). Beyond this limit, auditory events are perceived as being independent. When trying to synchronize taps to intervals longer than 1800 ms, adults tap after the onset of the target sound, in reaction to them rather than anticipating them. In other words, our study was aimed to examine children’s abilities to shift from rhythmic production, with short ITI, to interval production with long ITI (>1.5 s) in a sensorimotor synchronization task.

The existence of a common clock system for processing different temporal intervals is further supported by evidence on the temporal scalar property of estimations (Gibbon, 1977; Gibbon et al., 1984). Indeed, scalar timing is indexed by the increase in variability (SD) of temporal estimates as the duration of intervals (D) increases while the coefficient of variation of the temporal estimates (SD/D) remains constant for the different intervals values, as predicted by Weber’s law. However, an increasing number of studies suggest that the mechanisms underlying the processing of short intervals in the sub-second range are different from those underlying the processing of longer intervals. Neuroimaging studies for instance show that cerebral areas involved in the processing of short durations (<1 s) are different from those involved in the processing of long durations (Lewis and Miall, 2003; Grimm et al., 2004; Meck, 2005). In addition, several cases of violation of the temporal scalar property have been found when comparing short and longer durations in temporal reproduction tasks (Lejeune and Wearden, 2009). For instance, Bangert et al. (2011) showed a violation of the assumption of a single scalar timekeeper across millisecond and second timescales using a reproduction task with intervals from 300 to 1700 ms. Their results indeed suggest a shift in the region of 1 s for temporal reproduction. For tasks involving temporal production or reproduction, both Fraisse (1984) and Pöppel (1997) distinguish the perception of time for the processing of durations shorter than 1 s and time estimation for durations greater than 3 s, the latter requiring greater attention and different memory encoding. The *present*, according to Fraisse (1984) exists between both these two types of intervals. If there is a common mechanism for the production of short and long durations, we expect children to succeed in learning to synchronize their taps to longer ISI (>1700 ms). We thus hypothesize that children are able to switch from rhythm production to temporal interval production. Additionally, we expect children’s performance to improve with age as developmental studies have demonstrated an improvement with age in time sensitivity (for a review see, Droit-Volet, 2013; Vicario, 2013). However, if the mechanisms for short and long intervals are different, we expect that children would never succeed in producing long ITI in a temporal synchronization task despite the training sessions and regardless of their ages. Their motor rhythm would remain closed to their preferred tempo at both ages. Obviously, this does not mean that children are unable to produce or discriminate short durations (Zélanti and Droit-Volet, 2011), but that the production of ITI in the sensorimotor synchronization task involves timing processes specific to this task related to the production of temporal rhythm.

The aim of this study was thus to examine whether 3- and 5-year-old children with similar SMT can learn to increase the length of their ITI in a synchronization task. The children were asked to tap in synchrony with a 200 ms auditory sound. The ISI value progressively increased from session to session from 400 ms, close to children’s referent period, to longer periods: 630, 1000, 1600, 2500, and 4000 ms. The motor tempo produced by the children without the auditory stimuli was also assessed before and after this synchronization phase (respectively called pre-synchronization phase and post-synchronization phase). In each synchronization phase, the children received reinforcement when their taps occurred during the auditory stimulus or just before (15% of the ISI duration before). In addition, a group of control children were given the synchronization task with longer durations of 4 s for all sessions, in other to examine whether children directly succeed in learning to produce long ITI, without a period of learning based on the gradual modification of their initial SMT.

## MATERIALS AND METHODS

### PARTICIPANTS

Forty children took part in this experiment: twenty 3-year-olds (10 girls and 10 boys; mean age = 3.33, SD = 0.02) and twenty 5-year-olds (10 girls and 10 boys; mean age = 5.42, SD = 0.02). These children were recruited in a nursery school in Paris, France. The parents, the school director and the representative of the government of the French National Education signed a consent form for the participation of children in this experiment following the ethical principles of the Declaration of Helsinki. Only children who were volunteers participated in this experiment.

### APPARATUS

Children were tested individually in a quiet room in their school. They were seated in front of an inclined 14-inch computer monitor covered by a transparent Plexiglas plate. Children were asked to produce taps on this plate with their preferred hand. A computer recorded each tap via a high sensitive pressure transducer placed between the plate and the monitor. Each tap was recorded by the computer with a temporal precision of 1 ms. The pressure transducer sensitivity was very low in order to record both slight fingertip taps and strong taps. The auditory stimulus was an animal squeal produced by the computer speaker. The duration of the squeal was 200 ms. There were twelve different squeals (dog, cat, sparrow, duck, frog, monkey, bee, bear, hen, seal, pigeon, pup), all recorded with the same intensity. When the ITI was “correct,” it was followed by a feedback presented in the center of the computer screen, in the form of the picture (10 cm × 15 cm) of the animal corresponding to the heard squeal. The animal squeal changed after every set of 20 taps (**Table 1**).

**Table 1:** Procedure for the 0.4/4.0-s ISI group and the 4.0-s ISI group.

	Pre-synchronization	Synchronization	Post-synchronization
	
	20 ITI	Eight sets of 20 ISI	20 ITI
**Session 1**			
0.4/4.0-s group	Reinforced ITI at 4.0 s	0.4-s ISI	Reinforced ITI at 0.4 s
4.0-s group	Reinforced ITI at 4.0 s	4.0-s ISI	Reinforced ITI at 4.0 s
**Session 2**
0.4/4.0-s group	Reinforced ITI at 0.4 s	0.63-s ISI	Reinforced ITI at 0.63 s
4.0-s group	Reinforced ITI at 4.0 s	4.0-s ISI	Reinforced ITI at 4.0 s
**Session 3**
0.4/4.0-s group	Reinforced ITI at 0.63 s	1.0-s ISI	Reinforced ITI at 1.0 s
4.0-s group	Reinforced ITI at 4.0 s	4.0-s ISI	Reinforced ITI at 4.0 s
**Session 4**
0.4/4.0-s group	Reinforced ITI at 1.0 s	1.6-s ISI	Reinforced ITI at 1.6 s
4.0-s group	Reinforced ITI at 4.0 s	4.0-s ISI	Reinforced ITI at 4.0 s
**Session 5**
0.4/4.0-s group	Reinforced ITI at 1.6 s	2.5-s ISI	Reinforced ITI at 2.5 s
4.0-s group	Reinforced ITI at 4.0 s	4.0-s ISI	Reinforced ITI at 4.0 s
**Session 6**
0.4/4.0-s group	Reinforced ITI at 2.5 s	4.0-s ISI	Reinforced ITI at 4.0 s
4.0-s group	Reinforced ITI at 4.0 s	4.0-s ISI	Reinforced ITI at 4.0 s

### PROCEDURE

The children were randomly assigned to a “4.0-s” and a “0.4/4.0-s” group. In each group, the children were given six sessions of synchronization, one session per day. In the 4.0-s group, the ISI was always 4.0 s (**Table 1**). In the 0.4/4.0-s group, the ISI progressively increased from one session to the next: 0.4 s (session 1), 0.63 s (session 2), 1.0 s (session 3), 1.6 s (session 4), 2.5 s (session 5), 4.0 s (session 6). Each session was composed of three successive phases: (1) a pre-synchronization phase, (2) a synchronization phase, (3) and a post-synchronization phase. In the synchronization phase, the children were given eight successive sets of 20 auditory ISI. The children were asked to tap at the same time as the auditory stimulus in order to activate the pictures (feedback). When the child’s response occurred within a temporal window ranging from 15% before the auditory stimulus onset until the auditory stimulus offset, the ITI was considered “correct” and the visual feedback is provided immediately. However, for the responses given in the period before the stimulus onset, the visual feedback was delivered at the same time as the auditory stimulus. In addition, the duration of the feedback presentation did not exceed that of the auditory stimulus. Its duration therefore decreased with the increase of the delay between the child’s response and the auditory stimulus onset. In both the pre-synchronization and the post-synchronization phase, 20 ITI were recorded. In these phases, the procedure was similar to that used for the synchronization phase, except that the children did not hear the auditory stimulus. The post-phase was quite similar to the continuation phase usually used in rhythmic tasks (except that visual feedback was given, as explained previously). In contrast, the pre-synchronization phase assessed the children’s SMT for the first session, and the capability of recalling the ISI presented in the previous session. The children were thus told that they must try to get pictures by tapping at the right rhythm. Children were offered a demonstration trial by the experimenter prior to the synchronization phase of the first session.

## RESULTS

For the synchronization, pre-synchronization and post-synchronization phases, three indexes of performance were measured: the median ITI, the coefficient of variation of ITI (Q3-Q1/median^∗^100), and the percentage of reinforced ITI, i.e., followed by a feedback.

### SYNCHRONIZATION PHASE

For each index of performance, an analysis of variance (ANOVA) was conducted with three between-subjects factors (age, group, sex) and two within-subjects factors (synchronization sets, session). When Mauchly’s sphericity test indicated that the assumption of sphericity had been violated for stimulus duration, the degrees of freedom were adjusted using the Greenhouse–Geisser correction in order to take this violation into account in the statistical analysis.

#### Median ITI

**FIGURE 1:**
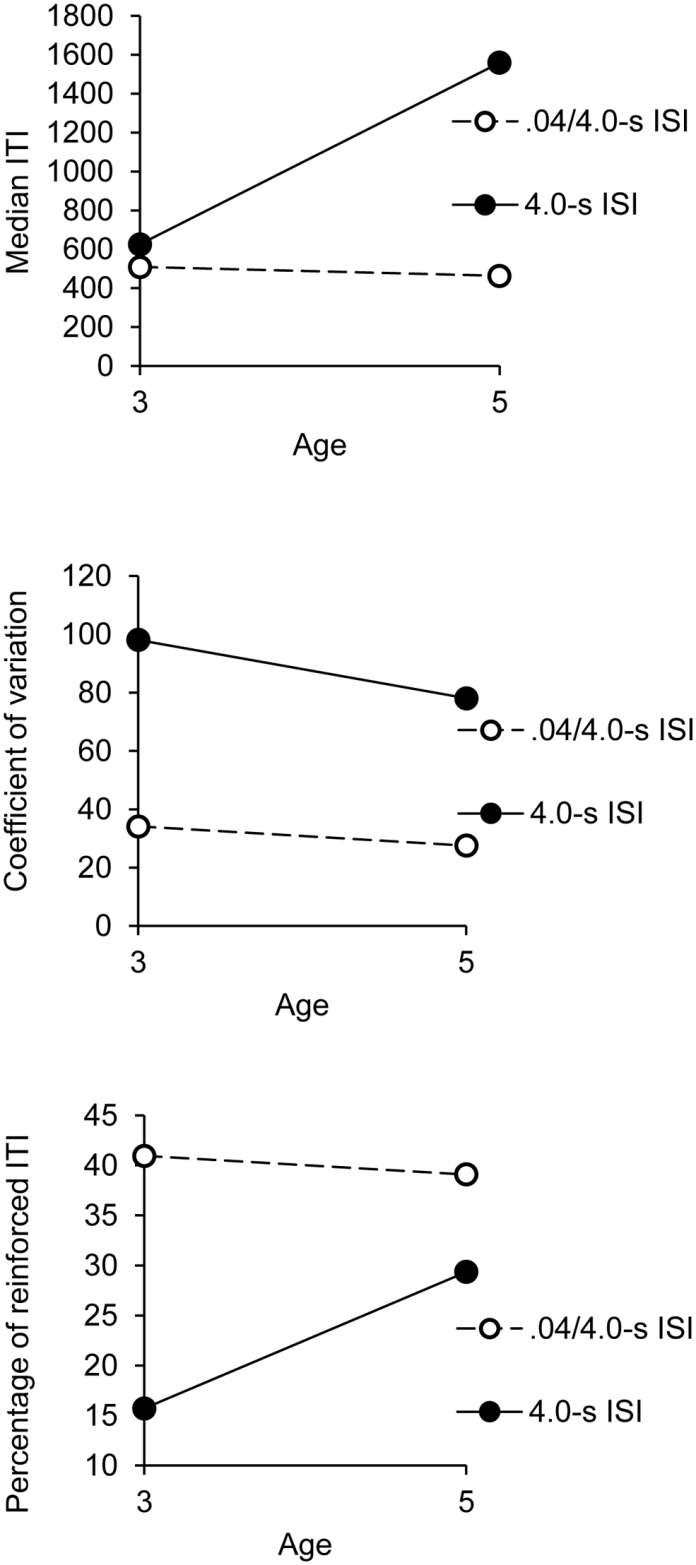
**Synchronization phase: indexes of performance as a function of age and group**.

The ANOVA showed a significant interaction between age and group, *F*(1,32) = 4.55, *p* = 0.04, $\eta_{p}^{2}$ = 0.12, with a significant main effect of group, *F*(1,32) = 6.96, *p* = 0.01, $\eta_{p}^{2}$ = 0.18, while the main effect of age failed to reach significance, *F*(1,32) = 3.73, *p*= 0.06. Age did not interact with any other factor. This significant interaction is illustrated in **Figure 1**. As shown in **Figure 1** (upper panel), the 5-year-olds succeeded in lengthening the median of their ITI in the 4.0-s ISI condition compared to the 0.4/4.0-s ISI condition, *t*(18) = 2.55, *p* = 0.02, while the 3-year-old obtained the same median ITI in these two temporal conditions, *t*(18) = 1.60, *p* = 0.13.

Consequently, the 5-year-olds produced longer ITI than did the 3-year-olds in the 4.0-s group, *t*(18) = 2.17, *p* = 0.04. However, in the 0.4/4.0-s ISI condition, when the initial rhythm was close to children’s spontaneous motor tempo, no difference was observed between the 3- (*M* = 508, SE = 218) and the 5-year-olds (*M* = 463, SE = 218), *t*(18) = 0.61, *p* = 0.55, irrespective of the different synchronization sessions. In this 0.4/4.0-s ISI condition, the mean of median ITI produced during the synchronization phase of all the sessions was close to 0.5 s.

**FIGURE 2:**
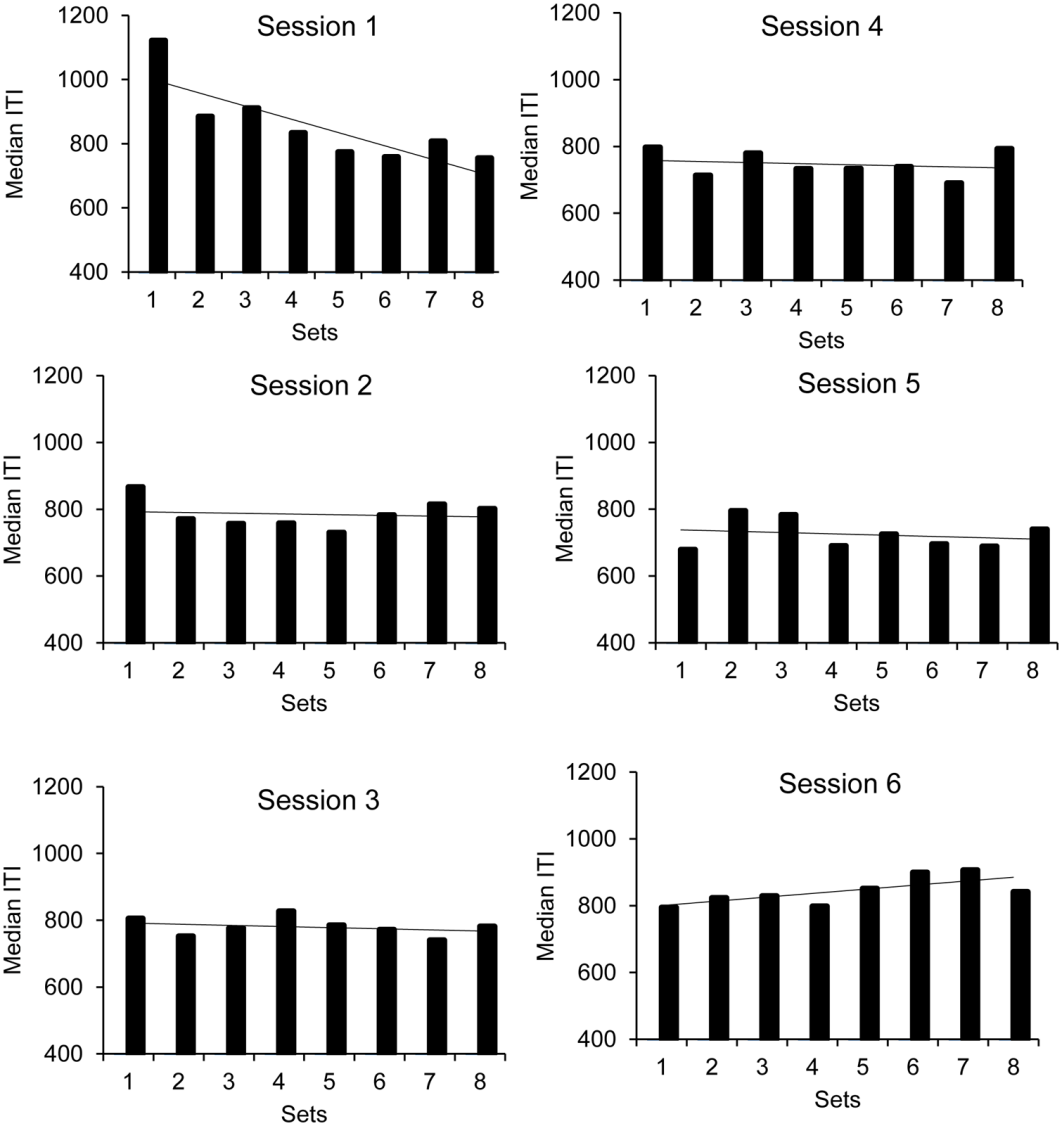
**Synchronization phase: median ITI as a function of sessions and sets**.

The ANOVA also showed a main effect of set, *F*(7,224) = 2.43, *p* = 0.02, $\eta_{p}^{2}$ = 0.07, as well as a significant set × session interaction, *F*(35,1120) = 2.36, *p* = 0.0001, $\eta_{p}^{2}$ = 0.07, the main effect of session being non-significant, *F*(5,160) = 1.15, *p* = 0.34. The set or the session factor did not interact with the group factor. To analyze this significant interaction, we ran an ANOVA on the median ITI with ‘set’ as within-subjects factor for each session taken separately (**Figure 2**, upper panel). The results showed a significant effect of set only for the first session, *F*(7,273) = 6.14, *p* = 0.0001. The effect of set did not reach significance for any of the other sessions (all *p* > 0.05). As shown in **Figure 2**, for the first session, there was a significant linear decrease in the median ITI from the first to the eighth set of the synchronization phase, *F*(1,39) = 10.76, *p* = 0.002, indicating that the children speeded up their motor tapping under the effect of rhythmic auditory stimuli.

The overall ANOVA also showed a significant interaction between sex and session, *F*(5,160) = 3.37, *p* = 0.006, $\eta_{p}^{2}$ = 0.10, with no main effect of sex, *F*(1,32) = 0.01, *p* = 0.91. Indeed, the boys progressively speeded up the rhythm of their tapping over sessions, as indicated by the significant linear session effect, *F*(5,95) = 4.98, *p* = 0.03, whereas the girls tended to maintain their tapping rhythm over sessions, *F*(5,95) = 1.95, *p* = 0.09.

#### Coefficient of variation of ITI

The ANOVA on the coefficient of variation showed a significant main effect of group, *F*(1,32) = 17.16, *p* = 0.0001, $\eta_{p}^{2}$ = 0.35, though group did not interact with any other factor (all *p* > 0.05). This indicated that the children produced more variable ITI in the 4.0-s group than in the 0.4/4.0-s group, regardless of age and synchronization session (**Figure 1**, middle panel). The variability of ITI was thus greater when the children tended to synchronize their taps to longer ISI. The main effect of age was not significant, *F*(1,32) = 0.92, *p* = 0.34, but age significantly interacted with session, *F*(5,160) = 2.99, *p* = 0.01, $\eta_{p}^{2}$ = 0.09. Three and 5-year-olds thus produced or tended to produce more variable ITI in the 4.0-s group than in the 0.4/4.0-s group [*t*(18) = 5.26, *p* = 0.0001, *t*(18) = 1.98, *p* = 0.06, respectively], but the between-group difference was greater for the 3-year-olds than for the 5-year-olds. There was also a significant sex × set interaction, *F*(7,224) = 2.84, *p* = 0.007, $\eta_{p}^{2}$ = 0.08. The other main effect or interactions were not significant. Consequently, the boys, who tended to produce shorter ITI than the girls (see above), obtained the same coefficient of variation of ITI over sessions, *F*(7,133) = 1.65, *p* = 0.13, whereas the variability of ITI tended to increase over sessions for the girls, *F*(7,133) = 1,93, *p* = 0.07, $\eta_{p}^{2}$ = 0.09.

#### Percentage of reinforced ITI

As for the median ITI, the ANOVA run on the percentage of reinforced ITI obtained during the synchronization phase showed a significant interaction between age and group, *F*(1,32) = 6.27, *p* = 0.02, $\eta_{p}^{2}$ = 0.16, with a significant main effect of group, *F*(1,32) = 31.86, *p* = 0.0001, $\eta_{p}^{2}$ = 0.50, and the main effect of age that tended toward significance, *F*(1,32) = 3.62, *p* = 0.07, $\eta_{p}^{2}$ = 0.10 (**Figure 1**). Consistently with the fact that the 5-year-olds produced longer ITI than did the 3-year-olds, the older children obtained a greater percentage of reinforced ITI than the younger ones in the 4.0-s group [20 vs. 16, *t*(18) = 2.54, *p* = 0.02; **Figure 1**, bottom panel]. However, in the 0.4/4.0-s group, when the children had to produce shorter ITI, the 5-year-olds obtained a percentage of reinforced ITI similar to the 3-year-olds,’ *t*(18) = 0.66, *p* = 0.52.

**FIGURE 3:**
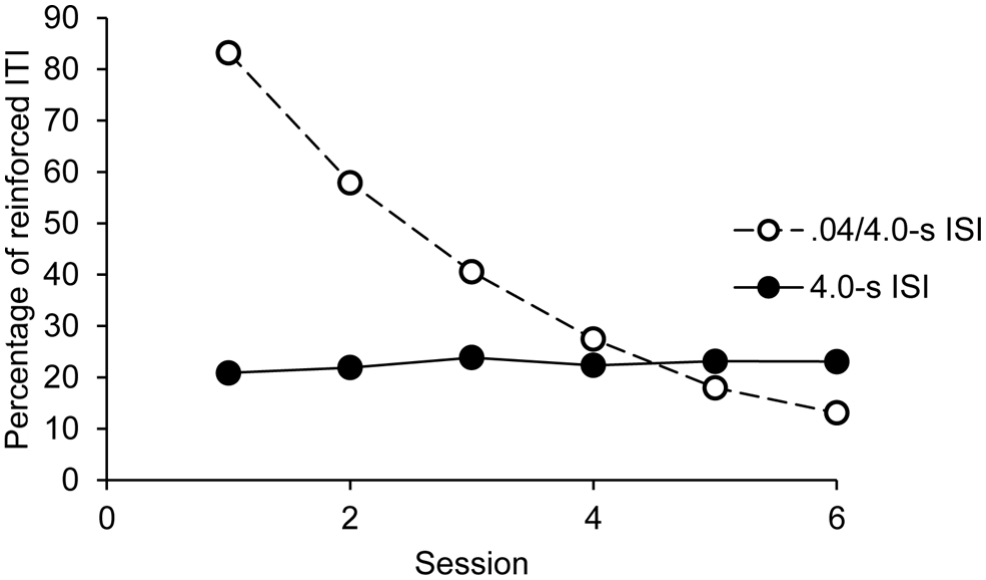
**Synchronization phase: percentage of reinforced ITI as a function of sessions and groups**.

The overall ANOVA also showed a significant main effect of session, *F*(5,160) = 129.61, *p* = 0.0001, $\eta_{p}^{2}$ = 0.80, and a significant session × group interaction, F(5,160) = 146.36, *p* = 0.0001, $\eta_{p}^{2}$ = 0.82. No other effect was significant. As illustrated in **Figure 3**, the percentage of reinforced ITI remained stable over the sessions in the 4.0-s ISI condition, *F*(5,95) = 0.64, *p* = 0.67, while it decreased in the 0.4-4.0-s ISI condition, *F*(5,95) = 212.23, *p* = 0.0001, $\eta_{p}^{2}$ = 0.92, from one session to the next (for all sessions comparisons with the Bonferroni test, *p* < 0.05).

### PRE-SYNCHRONIZATION AND POST-SYNCHRONIZATION PHASE

Using the same indexes as in the synchronization phase, an ANOVA was conducted with phase (pre-synchronization vs. post-synchronization phase) and session as within-subjects factors, and the same three between-subjects factors: age, group and sex. When Mauchly’s sphericity test indicated that the assumption of sphericity had been violated for stimulus duration, the degrees of freedom were adjusted using the Greenhouse–Geisser correction.

#### Median ITI

**FIGURE 4:**
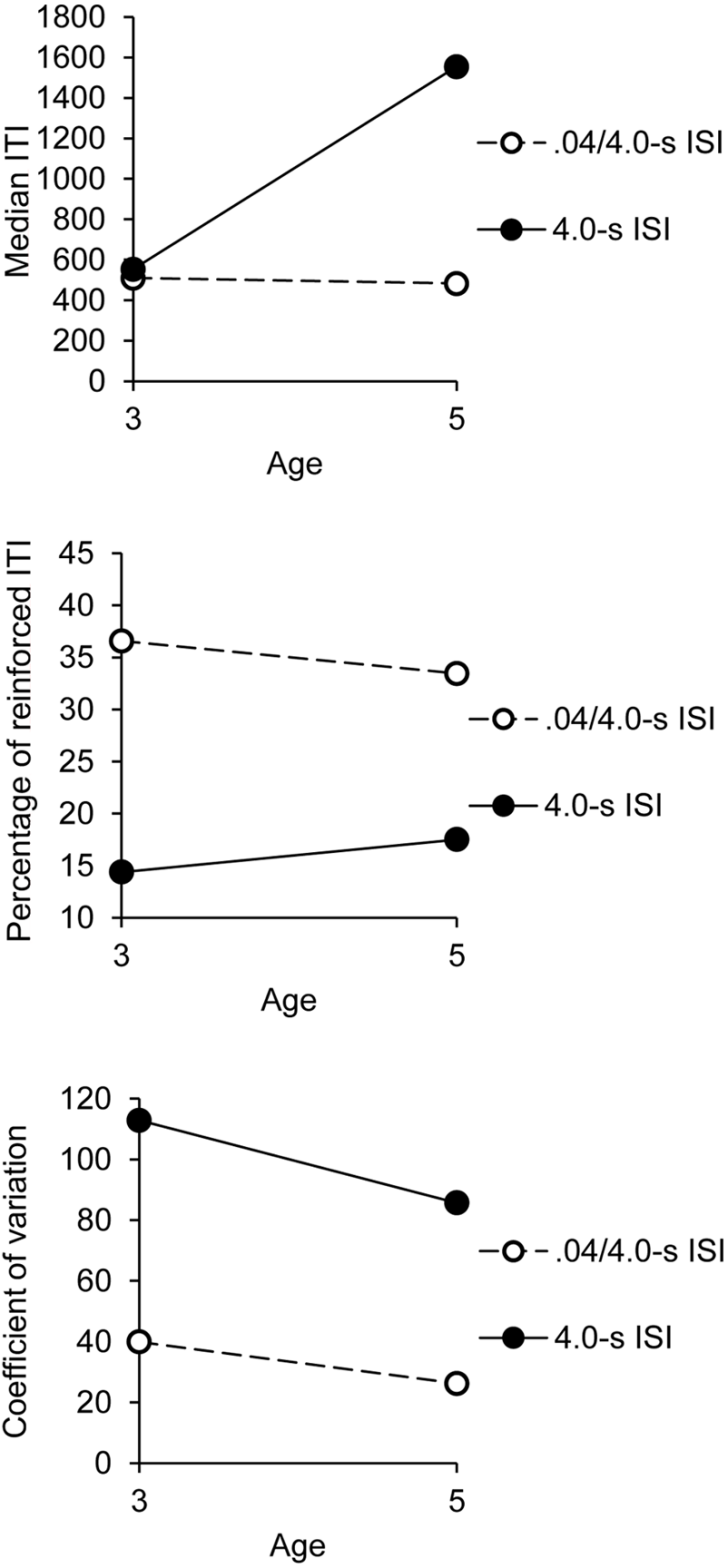
**Pre- and post-synchronization phases (averaged): indexes of performance as a function of age and group**.

As for the synchronization phase, there was a significant interaction between group and age, *F*(1,32) = 4.60, *p* = 0.04, $\eta_{p}^{2}$ = 0.13, with significant main effects of age, *F*(1,32) = 4.13, *p* = 0.05, $\eta_{p}^{2}$ = 0.11, and group, *F*(1,32) = 5.41, *p* = 0.03, $\eta_{p}^{2}$ = 0.15. As shown in **Figure 4** (upper panel), the median ITI was longer in the 4.0-s group than in 0.4-/4.0-s group for the 5-year-olds, *t*(18) = 2.38, *p* = 0.03, while the median ITI value did not change between groups for the 3-year-olds, *t*(18) = 0.62, *p* = 0.54. Consequently, the ITI was longer in the 5- than in the 3-year-olds in the 4.0-s group, *t*(18) = 2.22, *p* = 0.04, while it remained similar between the two age groups in the 0.4/4.0-s group, *t*(18) = 0.36, *p* = 0.72. No other effect was significant, except the main effect of phase, *F*(1,32) = 5.24, *p* = 0.03, $\eta_{p}^{2}$ = 0.14, indicating that the median ITI was shorter in the post- than in the pre-synchronization phase (830 vs. 722), irrespective of age. In other words, the synchronization task tended to speed up the initial motor rhythm.

#### Coefficient of variation of ITI

For the coefficient of variation, there was only a main effect of group, *F*(1,32) = 19.59, *p* = 0.0001, $\eta_{p}^{2}$ = 0.38, the main effect of age and the age × group interaction being non-significant (both *p* > 0.05; **Figure 4**, middle panel). Group did not significantly interact with other factors (all *p* > 0.05). The children thus produced more variable ITI in the 4.0-s ISI condition than in the 0.4/4.0-s ISI condition (99 vs. 33).

**FIGURE 5:**
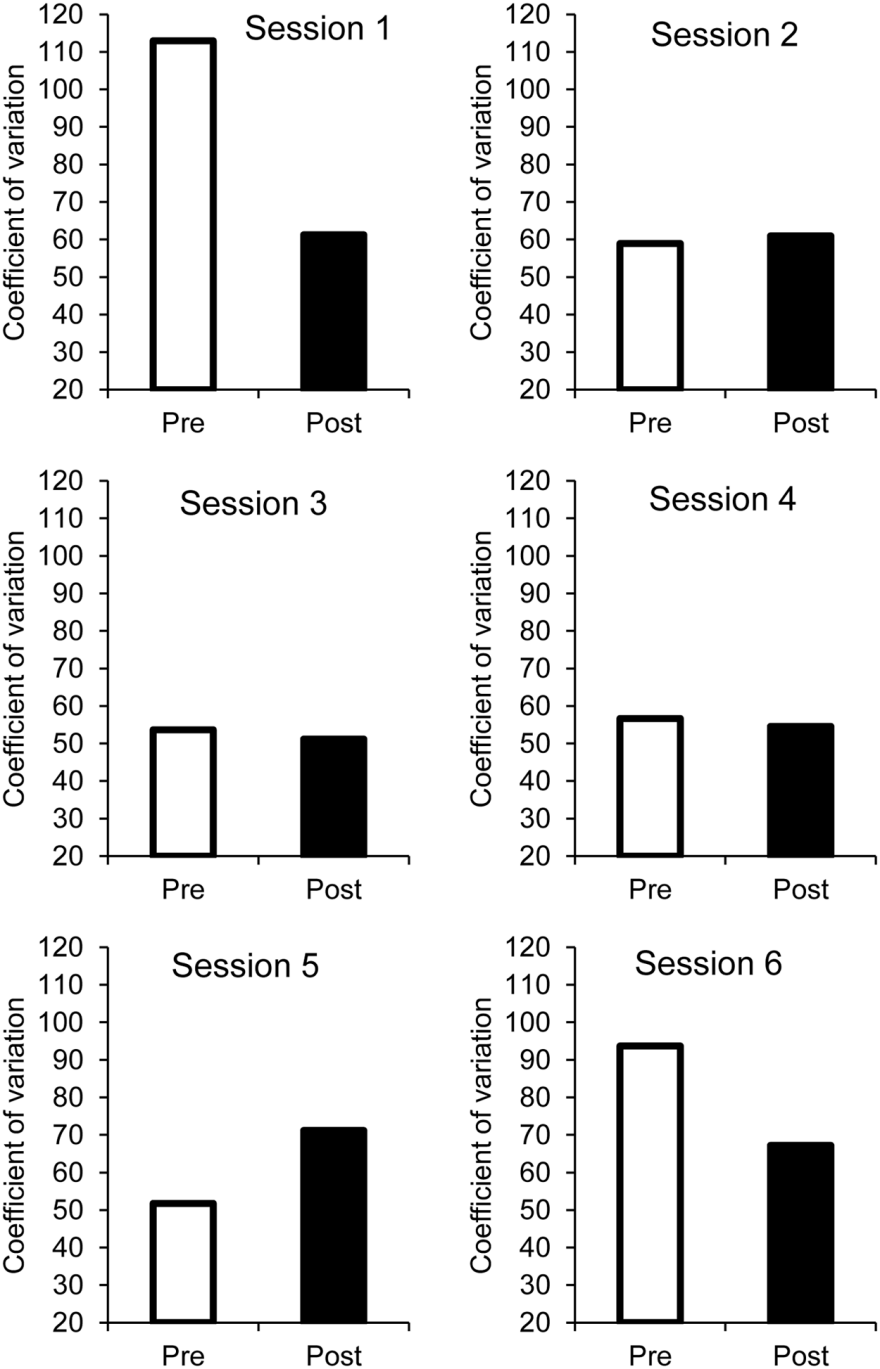
**Pre- and post-synchronization phases: coefficient of variation as a function of sessions**.

There was also a significant main effect of phase (pre- vs. post-synchronization phase), *F*(1,32) = 5.86, *p* = 0.02, $\eta_{p}^{2}$ = 0.16, as well as a significant phase × session interaction, *F*(5,160) = 3.13, *p* = 0.01, $\eta_{p}^{2}$ = 0.09, no other effect reached significance. The coefficient of variation of ITI (**Figure 5**) significantly decreased after the synchronization phase (Bonferroni tests, *p* < 0.05), especially for the latest session (session 6) with the longest ISI and for the first session, likely linked to the high level of inter-individual variability in coefficient of variation of ITI for these sessions.

#### Percentage of reinforced ITI during pre- and post-synchronization phase

**FIGURE 6:**
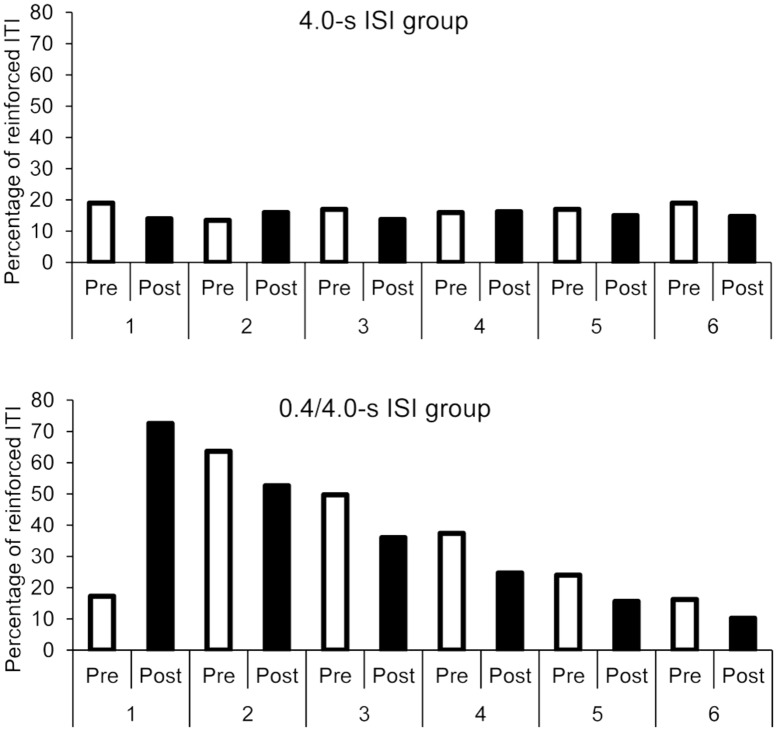
**Pre- and post-synchronization phases: comparison of the percentage of reinforced ITI as a function of groups and sessions**.

The overall ANOVA again showed a significant interaction between group and age, *F*(1,32) = 4.22, *p* = 0.048, $\eta_{p}^{2}$ = 0.117, with a significant main effect of group, *F*(1,32) = 157.30, *p* = 0.0001, $\eta_{p}^{2}$ = 0.83, but without significant main effect of age, *F*(1,32) = 0.01, *p* = 1.0. Concerning phase (pre- vs. post-synchronization phase), the participants thus obtained a greater percentage of reinforced ITI in the 0.4/4.0-s than in the 4.0-s group, but the magnitude of the between-group difference was lower for the 5-year-olds than for the 3-year-olds, this difference was due to the older children who tended to increase the length of their ITI. There was also a 3-way interaction between session, phase (pre- vs. post-synchronization phase) and group, *F*(5,160) = 61.13, *p* = 0.0001, $\eta_{p}^{2}$ = 0.66, with a significant session effect, *F*(5,160) = 75.12, *p* = 0.0001, $\eta_{p}^{2}$ = 0.70, session × group, *F*(5,160) = 86.13, *p* = 0.0001, $\eta_{p}^{2}$ = 0.73, phase × group, *F*(1,32) = 5.18, *p* = 0.03, $\eta_{p}^{2}$ = 0.14, and session × phase interaction, *F*(5,160) = 48.40, *p* = 0.0001, $\eta_{p}^{2}$ = 0.60. For each group taken separately (0.4/4.0-s and 4.0-s group), the ANOVA showed a significant phase (pre- vs. post-synchronization phase) × session interaction [*F*(5,95) = 76.08, *p* = 0.0001, $\eta_{p}^{2}$ = 0.80, *F*(5,95) = 2.40, *p* = 0.04, $\eta_{p}^{2}$ = 0.11, respectively]. As shown in **Figure 6**, in the 0.4/4.0-s group, participants obtained more reinforced ITI after (72%) than before (17%) the synchronization phase in the first session with the 0.4-s ISI (Bonferroni, *p* < 0.05). However, after this first session, the percentage of reinforced ITI systematically decreased after the synchronization phase whatever the values of ISI (for all sessions, *p* < 0.05), in such a way that the percentage of reinforced ITI for the post-synchronization phase in sessions 5 and 6 returned to the percentage obtained in the pre-synchronization phase for the first session (*p* > 0.05).

Nevertheless, the percentage of ITI obtained in the post-synchronization phase for the sessions before the fifth session was always greater than that obtained in the pre-synchronization phase of the first session (all *p* > 0.05), thereby suggesting a sort of temporal learning in children. For the 4.0-s group, the percentage of reinforced ITI remained low and did not significantly change between the post- and the pre-synchronization phase for all sessions (all *p* > 0.05), except for the first and the last session, for which a lower percentage of reinforced ITI for the post- than for the pre-synchronization phase was observed (*p* < 0.05). This attests to children’s difficulty in learning to produce long ITI in a synchronization task.

The overall ANOVA also revealed a significant sex × age × synchronization phase interaction, *F*(1,32) = 4.37, *p* = 0.045, $\eta_{p}^{2}$ = 0.12, with no other significant effect. This significant interaction indicated that, regardless of session, the percentage of reinforced ITI was lower in the post- than in the pre-synchronization for 5-year-old boys (25 vs. 23, Bonferroni test, *p* < 0.05), but not girls (0.26 vs. 0.27, *p* > 0.05). At three children showed, no significant between-phase difference in the percentage of reinforced ITI was as a function of sex (all *p* > 0.05).

## DISCUSSION

The purpose of this study was to further examine the characteristics of auditory-motor coordination during early childhood. Specifically we investigated whether, in a synchronization task, 3- and 5-year-olds children can learn to change their preferred tempo to produce ITI longer than 1 s, in other words to switch between rhythm production and temporal interval production.

Our results revealed that children succeed in synchronizing their taps with an external tempo when it is close to their own SMT, as indicated by the high percentage of reinforced ITI, as well as the median ITI (close to 400-ms) in the synchronization phase of the first session of the 0.4/4.0-s group. Consequently, children succeed in speeding up their tempo under the effect of an external rhythm with a 0.4-s ISI, as suggested by the progressive decrease of median ITI from an ISI set to another set in the synchronization phase of the first session. However, once this motor rhythm was initiated, the children were not successful in changing it, i.e., in slowing down their motor rhythm in the synchronization sessions with longer ITI. In the 0.4/4.0-s condition, the percentage of reinforced ITI indeed decreased across sessions as the length of ISI increased (**Figure 3**), while the median ITI did not vary. In addition, beyond a threshold of 1.0-s (session 3), when ISI was too far away from their own SMT, the percentage of reinforced ITI fell below 50%. This means that less than one tap out of two was reinforced. Further experiments are required to examine whether the children could learn to progressively produce longer ITI in a synchronization task with more training sessions and smaller ISI differences between sessions that those used in our study. Nevertheless, our data suggest a lack of flexibility in sensory-motor synchronization in young children, at least in children aged 3- and 5-year-olds. This is consistent with the results of studies using short ITIs (<900-ms), reported in the introduction, showing that young children are able to synchronize their taps to an external auditory tempo but only when it was close to their own tempo (Provasi and Bobin-Bègue, 2003; Bobin-Bègue and Provasi, 2008). For instance, Provasi and Bobin-Bègue (2003) showed that the 2^1^/_2_ – and 4-year-old children, who had to synchronize their tapping rhythm to an 800 ms auditory tempo, maintained a spontaneous tapping rhythm of around 400 ms.

The question raised here is: what factor explains this lack of flexibility in sensory-motor synchronization in young children. Are factors related to the development of motor and cognitive capacities during childhood or factors related to the specificity of temporal mechanisms underlying rhythmical activities, which would radically differ from those involved in the production of temporal interval, or both? Some results in our study suggest that developmental factors account in part of this lack of flexibility in children’s synchronization behavior. Indeed, in the 4.0-s condition, when the children must directly learn to synchronize their taps to a 4.0-s ISI, the 5-year-old children succeeded in lengthening their ITI compared to the younger children. In the different synchronization phases (pre-synchronization and post-synchronization phases and synchronization phase *per se*), the 5-year-olds indeed obtained longer median ITI than did the 3-year-olds and the percentage of reinforced ITI was higher for the 5-year-olds than for the 3-year-olds. The difference in the coefficient of variation of ITI between the groups was also more important for the 3-year-olds than for the 5-year-olds. The ITI were thus more variable in the 4.0-s condition than in the 0.4/4.0-s group, especially for the 3-year-olds. In summary, this confirmed our hypothesis that with as they develop, children learn to produce long intervals in a synchronization task.

The developmental sources of this age-related change in performance in a synchronization task are far from being clear. In a recent study however, Provasi et al. (2014) used neuropsychological tests to assess the cognitive and fine motor skills of children aged between 5 and 14 performing a synchronization task with ITI of 400-ms and 600-ms. They found that age-related increase in both fine motor skills and speed of processing were the best predictors of individual variance in children’s synchronization performance. Synchronization performance thus improves with the development of abilities involved in the rapid production of motor acts under temporal constraints. In addition, in our study, the children were required to inhibit a spontaneous motor behavior to produce longer intervals. It has been clearly demonstrated that it is particularly difficult for children to slow down their own tapping rhythm and/or to inhibit the triggering of a tap (Hulsebus, 1973; Condon and Sander, 1974). This is explained by the limited inhibitory capacities in young children related to the slow maturation of the prefrontal cortex involved in attention capacities (see e.g., Bjorklund and Harnishfeger, 1995; Rubia, 2013). In the same way, Bobin-Bègue and Provasi (2008) found that the 3-year-olds children were able to accelerate their tapping rhythm during a synchronization task but they did not slow down their tapping rhythm when the auditory tempo was slower than their own spontaneous motor tempo. Consequently, the development of cognitive control capacities (motor inhibition), information processing speed) and fine motor skills could explain the age-related improvement in performance in a sensorimotor synchronization task.

However, in our study, even in the 4.0-s group, when the 5-year-olds succeeded in lengthening their ITI, the value of their ITI did not exceed 1500 ms. The mean of their median ITI was indeed 1559 ms for the different synchronization sessions. This ITI value is remarkable because it is very close to the temporal limit in the production of rhythms pointed out in various studies (Fraisse, 1984; Repp, 2006; Bangert et al., 2011). As explained by Repp (2006), when a tempo is longer than 1800 ms, adults perceive auditory events as independent events, and their responses occur after the beginning of the auditory event, rather than before the auditory events as an anticipation. Our results thus suggest that specific mechanisms underlying the production of rhythm also explain the lack of flexibility of sensorimotor synchronization behaviors observed in children. Our result point to children’s difficulty, even impossibility, in switching from rhythmic production to interval production with long interval (>1.5 s) in a synchronization task. This does not mean that children are unable to produce long intervals. Several studies using a fixed interval (FI) schedule of reinforcement have demonstrated that young children can learn to produce long intervals between two reinforced responses (for a review see Droit-Volet et al., 2005; Droit-Volet, 2011, 2013). Our study suggests rather that rhythmic activities involve different mechanisms for the production of ITI shorter and longer than 1-2 s. This is consistent with recent data from adults suggesting there are two processes within this range of durations (Rammsayer and Troche, 2014). This is also consistent with results from neuroimaging studies suggesting that the activation of the cerebellum would be restricted to timing of action in the range of temporal intervals shorter than 1 s (Ivry et al., 2002; Wiener et al., 2010). In summary, our study provides empirical data confirming a border around 1 and 2 s between different temporal processes in a synchronization task, beyond which rhythm is lost.

Furthermore, although there is no main effect of sex on synchronization performance in our study, our results indicate that boys progressively speed up their tapping rhythm through sessions in the synchronization phase whereas girls maintain their tapping rhythm. Indeed, the percentage of reinforced ITI was lower in the post-synchronization phase than in the pre-synchronization phase for boys, but not for girls. However, this sex difference in synchronization performance was only observed at the age of 5 years. This suggests that, at that age, when children begun to succeed in changing their tapping rhythm, boys had more difficulties to do so than girls. However a previous study conducted by Provasi and Bobin-Bègue (2003) showed that boys aged 4 years performed better than girls whatever the ISI (0.4, 0.6, and 0.8 s). Other experiments, with more participants, have therefore to be conducted on the differences between boys and girls in the development of rhythmic activities. There are indeed very few studies on the gender differences in cognitive development. However, some recent fMRI studies testing girls and boys across the age range from 7 to 38 years suggest a more mature activation in the fronto-striatal region during attentional control tasks (inhibition, switching) for girls than for boys (Christakou et al., 2009; Rubia et al., 2013; for a review see Rubia, 2013). Higher abilities in attentional control for girls than for boys related to the brain maturation might thus account for sex differences in synchronization observed in our study. However, there is also greater social pressure in education for women than men as regards inhibition of emotion and social responses. This education might thus also allow young girls to enhance their inhibition abilities compared to boys (Bjorklund and Kipp, 1996).

In conclusion, the present study demonstrates that young children do not easily learn to produce intervals longer than 1.5 s in a sensorimotor synchronization task, even with a procedure using visual feedback and several training sessions. Indeed, our results show that children do not succeed in switching from the production of rhythm to the production of temporal interval. The processing of short (<1.5 s) and long (>1.5 s) intervals would thus involve different mechanisms even in a same sensorimotor synchronization task. Nevertheless, 5-year-olds perform better than 3-year-olds revealing an age-related improvement in the flexibility of synchronization behavior. Further experiments are required with older children to better describe the development of this synchronization ability.

### Conflict of Interest Statement

The Guest Associate Editor Rana Esseily declares that, despite being affiliated to the same institution as author Anne Bobin-Bègue, the review process was handled objectively and no conflict of interest exists. The authors declare that the research was conducted in the absence of any commercial or financial relationships that could be construed as a potential conflict of interest.
